# Prolonged Excretion of Poliovirus among Individuals with Primary Immunodeficiency Disorder: An Analysis of the World Health Organization Registry

**DOI:** 10.3389/fimmu.2017.01103

**Published:** 2017-09-25

**Authors:** Grace Macklin, Yi Liao, Marina Takane, Kathleen Dooling, Stuart Gilmour, Ondrej Mach, Olen M. Kew, Roland W. Sutter, The iVDPV Working Group, Ousmane Diop

**Affiliations:** ^1^World Health Organization, Geneva, Switzerland; ^2^University of Tokyo, Tokyo, Japan; ^3^Centers for Disease Control and Prevention, Atlanta, GA, United States; ^4^Taskforce for Child Health, Atlanta, GA, United States

**Keywords:** polio eradication, primary immunodeficiency, vaccine-derived poliovirus, oral poliovirus vaccine, immunodeficiency-related vaccine-derived poliovirus

## Abstract

Individuals with primary immunodeficiency disorder may excrete poliovirus for extended periods and will constitute the only remaining reservoir of virus after eradication and withdrawal of oral poliovirus vaccine. Here, we analyzed the epidemiology of prolonged and chronic immunodeficiency-related vaccine-derived poliovirus cases in a registry maintained by the World Health Organization, to identify risk factors and determine the length of excretion. Between 1962 and 2016, there were 101 cases, with 94/101 (93%) prolonged excretors and 7/101 (7%) chronic excretors. We documented an increase in incidence in recent decades, with a shift toward middle-income countries, and a predominance of poliovirus type 2 in 73/101 (72%) cases. The median length of excretion was 1.3 years (95% confidence interval: 1.0, 1.4) and 90% of individuals stopped excreting after 3.7 years. Common variable immunodeficiency syndrome and residence in high-income countries were risk factors for long-term excretion. The changing epidemiology of cases, manifested by the greater incidence in recent decades and a shift to from high- to middle-income countries, highlights the expanding risk of poliovirus transmission after oral poliovirus vaccine cessation. To better quantify and reduce this risk, more sensitive surveillance and effective antiviral therapies are needed.

## Introduction

Since the launch of the Global Polio Eradication Initiative in 1988, there has been substantial progress toward eradication, documented by a decline in the incidence of cases from >350,000 in 1988 to 37 in 2016, and a decrease in the number of endemic countries from 125 to 3 (1). Although wild poliovirus type 1 continues to circulate in Afghanistan, Nigeria, and Pakistan, no wild poliovirus type 2 or wild poliovirus type 3 have been detected globally since October 1999 (2) and November 2012 (3), periods of 17 and 4 years, respectively.

These achievements have been accomplished through the extensive use of oral poliovirus vaccines (OPV), which are easy to administer, suitable for mass campaigns, and able to induce both humoral and mucosal immunity (4). However, in rare instances, live Sabin strains contained in OPV can cause vaccine-associated paralytic poliomyelitis, with paralytic manifestations indistinguishable from those caused by wild poliovirus (4). Furthermore, these viruses have the potential to revert to neurovirulence and re-acquire the transmissibility characteristics of wild poliovirus, resulting in outbreaks of circulating vaccine-derived poliovirus (VDPV) (5). In the presence of high population immunity, VDPVs rarely emerge and cause outbreaks. However, in areas with low population immunity, these viruses could potentially re-establish endemic transmission (6).

Immunodeficiency-related vaccine-derived poliovirus (iVDPV) is a type of VDPV in which individuals with a primary immunodeficiency disorder (PID) excrete Sabin polioviruses; in some cases, for substantially longer periods than immunocompetent individuals (4). After exposure to OPV, immunocompetent individuals usually excrete the vaccine virus for 4–8 weeks (7). However, in immunodeficient individuals, an inability to mount an adequate immune response can result in persistence of the intestinal infection with poliovirus and prolonged viral shedding (8). In the process, the virus can mutate to re-acquire the neurovirulence and transmissibility characteristics of wild poliovirus (9–12).

To address the risks associated with OPV use, the Strategic Plan of Action 2013–2018 of the Global Polio Eradication Initiative calls for a sequential global withdrawal of OPV, starting with the Sabin type 2 component that was removed in April 2016 and followed by Sabin type 1 and Sabin type 3 after certification of wild type 1 and 3 eradication, respectively (13). Although this strategy may minimize the long-term risk of circulating VDPV outbreaks, iVDPV cases will remain a potential source of live poliovirus in communities after the withdrawal of OPV and in the post eradication era, and could pose a substantial risk of poliovirus reintroduction in the population. The post-OPV cessation risks of long-term excretors have been modeled previously (14–16).

A number of case reports and case series of iVDPV excretors have been published with a systematic review of published cases undertaken (17), and more recently, studies have assessed iVDPV excretion among persons with PIDs (18–20). However, many of these works focus on only a subset of iVDPV cases, with case reports often limited to upper-middle income and high-income countries and patients with paralysis.

We present analysis of iVDPV cases known to the World Health Organization (WHO), from the inception of widespread OPV use (1962–2016). The main objectives of this study are to conduct demographic, risk factor, and survival analysis of reported iVDPV cases to determine the populations most at risk and to better understand the threat posed by iVDPV cases to global poliovirus eradication.

## Materials and Methods

### Study Population

This study analyzed cases in the iVDPV case registry maintained by WHO, containing reported cases from 1962 to 2016, irrespective of reporting source or paralysis status (see Table S2 in Supplementary Table). The main sources contributing cases to the registry include: (1) the acute flaccid paralysis (AFP) surveillance system, which is the routine way countries report cases; (2) the regional polio laboratory network; (3) specific studies and pilot poliovirus surveillance projects targeting PID patients; and (4) regularly conducted systematic literature reviews of scientific journals reporting on iVDPV cases, where key search terms include VDPV, iVDPV, immunodeficiency, immunocomprised, excretion, polio, and “vaccine-associated paralytic poliomyelitis.” In order to obtain the most recent information on ongoing cases, corresponding authors of case reports and/or relevant scientific and medical personnel, including regional laboratory coordinators, were contacted.

Inclusion criteria comprised: excretion of poliovirus for greater than 6 months; confirmed PID; and laboratory-confirmed VDPV. A *prolonged excretor* was defined as a person excreting virus for ≥6 months and ≤5 years, and a *chronic excretor* was defined as excreting for >5 years (14).

Primary immunodeficiency disorder in the registry includes individuals with congenital onset of B-cell deficiency, T-cell deficiency, major histocompatibility complex deficiency, or a combination of the above. For the purposes of these analyses, immunodeficiency disorders were divided into the following broad categories:

- Antibody disorder, including hypogammaglobulinemia, agammaglobulinemia, X-linked agammaglobulinemia, and other antibody deficiencies.
- Severe combined immunodeficiency disorder and other combined humoral/T-cell deficiencies.
- Common variable immunodeficiency disorder (CVID).
- Other, including major histocompatibility complex deficiencies, the immunodeficiency, centromere instability, and facial anomalies syndrome, as well as other unknown or undiagnosed causes of immunodeficiency.

### Laboratory Processing of Poliovirus Isolates

Until the establishment of sensitive surveillance for polio eradication, viruses were mainly isolated in a few specialized laboratories. With implementation of surveillance for polio eradication, poliovirus was increasingly isolated from patient stool samples by laboratories participating in the WHO Global Polio Laboratory Network. Isolates were identified by several steps: first, specimens were grown in tissue culture; second, molecular methods were used to identify the serotype; and third, intratypic differentiation to determine vaccine or wild strains was done either by enzyme-linked immunosorbent assay using highly specific cross-absorbed antisera and/or by diagnostic reverse transcriptase-polymerase chain reaction (21). If the enzyme-linked immunosorbent assay and molecular intratypic differentiation methods yielded discordant results, the ~900-nucleotide interval encoding the major capsid protein, the viral protein 1 (VP1), was sequenced. Poliovirus genomes appear to evolve at a rate of ~1.1% mutations in VP1 region per year (22) and VDPVs are defined as having a VP1 nucleotide divergence >1% for type 1 and 3, and >0.6% for type 2, from the corresponding parenteral OPV strain (23), consistent with prolonged replication or transmission.

### Demographic and Clinical Variables

Cases were recorded with the following demographic and clinical parameters: year of detection; country of residence and its income classification [based on the 2016 World Bank classifications (24)]; date of birth; date of onset; age at onset; gender; PID; presence or absence of paralysis; OPV vaccination history; clinical outcome (alive, alive and stopped excreting, dead or unknown); and first and most recent positive specimen dates, serotype of virus, percentage VP1 divergence from the parenteral Sabin strain, and recombination with other non-polio enteroviruses.

### Statistical Analysis

Descriptive analysis was conducted to identify demographic and clinical characteristics of iVDPV cases. According to available information, duration of excretion was estimated following the algorithm provided in Table S1 in Supplementary Material. The algorithm bases the length of excretion on either the time period between OPV administration (or exposure) and the last iVDPV isolate, the time period calculated by VP1 divergence from parental Sabin strain based on an evolution rate of ~1.1% divergence per year (22), or where this information was not available, the excretion time under observation. To model the length of excretion, Kaplan–Meier survival analysis was employed and log-rank tests used to determine difference in survival times between groups. To allow for multiple comparisons, the *P*-value for significance was set at *P* < 0.01. All analyses were conducted using the statistical software R 3.3.1 (25) and Stata MP 13 (26).

## Results

### Demographics

**Figure 1:**
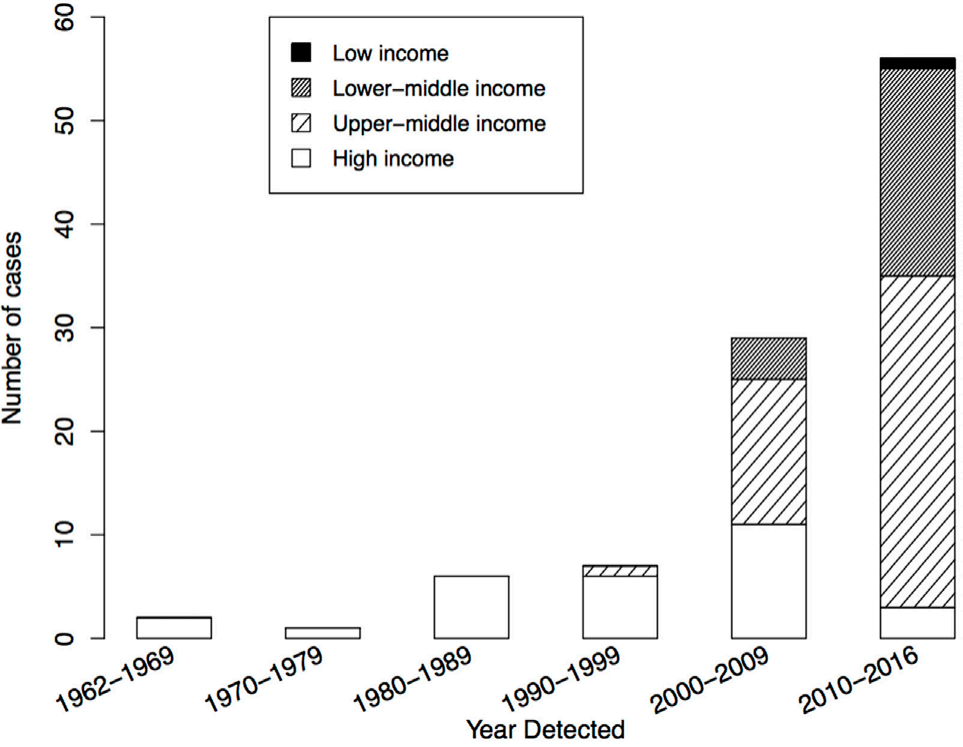
Year of detection of 101 reported chronic and prolonged immunodeficiency-related vaccine-derived poliovirus cases from 1962 to 2016, by income classification of country of residence: low income (*n* = 1), lower-middle income (*n* = 24), upper-middle income (*n* = 47), and high-income (*n* = 29). Income classification based on 2016 World Bank Classification.

As of July 2016, the WHO registry included 110 suspected iVDPV cases with onset of excretion or paralytic symptoms during 1962–2016. Of these, 101 (91.8%) met the definition of prolonged or chronic iVDPV excretion and were included in the analysis. Figure 1 shows the number of reported iVDPV cases over time since 1962. There has been an increase in the number of reported cases since the year 2000, with the highest number the period since 2010 (56/101). Within this, there are two divergent trends: an increase in cases reported from low- and middle-income countries and a decrease in the number of cases reported from high-income countries (Figure 1).

**Figure 2:**
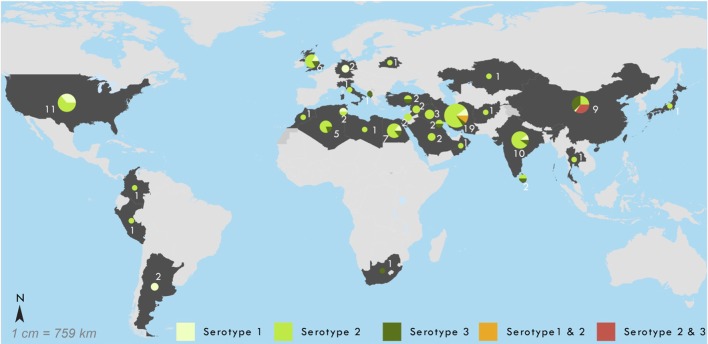
Geographic location of 101 reported chronic and prolonged immunodeficiency-related vaccine-derived poliovirus cases, 1962–2016. Shown by serotype of virus in most recent specimen available: 1 (*n* = 15), 2 (*n* = 68), 3 (*n* = 13), 1 + 2 (*n* = 2), and 2 + 3 (*n* = 3).

Figure 2 provides the geographic location of these cases by associated serotype, illustrating a large concentration of cases around the Middle East. The characteristics of the 101 prolonged and chronic iVDPV cases are presented in Table 1. The residence of cases by worldwide region shows the highest proportion of cases 43/101 (43%) occurred in the Eastern Mediterranean Region. There were 24/101 (24%) cases in lower-middle-income countries, 47/101 (47%) in upper-middle income countries, and 29/101 (29%) in high-income countries, with all seven chronic excretor cases resident in high-income countries. Only one case was reported from a low-income country, which was in Afghanistan in 2013.

**Table 1:** Baseline characteristics of 101 reported chronic and prolonged immunodeficiency-related vaccine-derived poliovirus cases at time of detection, 1962–2016.

Characteristic	Number of cases, *n*/*N* (%)	Prolonged excretor cases^a^, *n*/*N* (%)	Chronic excretor cases^b^, *n*/*N* (%)
Total	101	94	7

**Gender**			
Male	63/98 (64)	59/91 (65)	4/7 (57)
Female	35/98 (36)	32/91 (35)	3/7 (43)

**Age at onset or first positive specimen**			
<1 year	46/100 (46)	46/93 (49)	0/7 (0)
1 to <5 years	37/100 (37)	37/93 (40)	0/7 (0)
5 to <10 years	7/100 (7)	6/93 (6)	1/7 (14)
10 to <20 years	5/100 (5)	2/93 (2)	3/7 (43)
20 to <30 years	3/100 (3)	1/93 (1)	2/7 (29)
≥30 years	2/100 (2)	1/93 (1)	1/7 (14)

**Paralysis**			
Yes	71/100 (71)	67/93 (72)	4/7 (57)
No	29/100 (29)	26/93 (28)	3/7 (43)

**Country of residence income classification^c^**			
High income	29/101 (29)	22/94 (23)	7/7 (100)
Upper-middle income	47/101 (47)	47/94 (50)	0/7 (0)
Lower-middle income	24/101 (24)	24/94 (26)	0/7 (0)
Low income	1/101 (1)	1/94 (1)	0/7 (0)

**WHO region of residence**			
Eastern Mediterranean Region	43/101 (43)	43/94 (46)	0/7 (0)
African Region	6/101 (6)	6/94(6)	0/7 (0)
Western Pacific Region	10/101 (10)	7/94 (7)	3/7 (43)
European Region	14/101 (14)	10/94 (11)	4/7 (57)
South-East Asian Region	13/101 (13)	13/94 (14)	0/7 (0)
Region of the Americas	15/101 (15)	15/94 (16)	0/7 (0)

**Immunodeficiency disorder**			
SCID and other combined humoral/T-cell deficiencies	27/92 (29)	27/85 (32)	0/7 (0)
SCID	25/92 (27)	25/85 (29)	0/7 (0)
CVID	21/92 (23)	14/85 (16)	7/7 (100)
Antibody disorders	31/92 (33.7)	31/85 (36)	0/7 (0)
HGG	10/92 (11)	10/85 (12)	0/7 (0)
XLA	12/92 (13)	12/85 (14)	0/7 (0)
AGG	5/92 (5)	5/85 (6)	0/7 (0)
Other disorders	13/92 (14)	13/85 (15)	0/7 (0)
MHC II	6/92 (7)	6/85 (7)	0/7 (0)
HLA-DR	4/92 (4)	4/85 (5)	0/7 (0)

**Serotype^d^**			
1	15/101 (15)	12/94 (13)	3/7 (43)
2	68/101 (67)	64/94 (68)	4/7 (57)
3	13/101 (13)	13/94 (14)	0/7 (0)
1 + 2	2/101 (2)	2/94 (2)	0/7 (0)
2 + 3	3/101 (3)	3/94 (3)	0/7 (0)
All type 2 associated	73/101 (72)	69/94 (73)	4/7 (57)

**Outcome status**			
Dead	48/101 (48)	45/94 (48)	3/7 (43)
Stopped excreting	36/101 (36)	34/94 (36)	2/7 (29)
Alive (and excreting at last specimen)	8/101 (8)	7/94 (7)	1/7 (14)^e^
Unknown	9/101 (9)	8/94 (9)	1/7 (14)

*AGG, agammaglobulinemia; CVID, common variable immunodeficiency disorder; HGG, hypogammaglobulinemia; HLA-DR, human leukocyte antigen-antigen D related; MHC II; major histocompatibility complex type II; SCID, severe combined immunodeficiency disorder; WHO, World Health Organization; XLA, X-linked agammaglobulinemia*.

*^a^Defined as excreting virus for ≥6 months and ≤5 years*.

*^b^Defined as excreting for >5 years*.

*^c^Based on 2016 World Bank Classification*.

*^d^Serotype of virus in most recent specimen available*.

*^e^This case has been published (27)*.

Out of the 101 cases, 63/98 (64%) were males, 71/100 (71%) presented with paralytic manifestations, as defined by AFP, and the most common age of onset was <1 year in 46/100 (46%) cases, followed by 1 to <5 years old in 37/100 (37%) (Table 1).

### Underlying PID

The underlying immunodeficiency disorders of cases are shown in Table 1. Antibody disorders were the most frequent, accounting for 31/91 (34%) of the cases, primarily composed of hypogammaglobulinemia, agammaglobulinemia, and X-linked agammaglobulinemia. Severe combined immunodeficiency disorder and CVID were also common, present in 25/92 (27%) and 21/92 (23%) of cases, respectively. All the seven chronic excretors had CVID.

### Virological Factors

The serotype of excreted virus was predominantly type 2 poliovirus, in 68/101 cases (67%), followed by type 1 in 15/101 cases (15%) and type 3 in 13/101 cases (13%) (Table 1). Among the six cases with multi-serotype infections, two were type 1 and 2 co-infections and three were type 2 and 3 co-infections, resulting in 73/101 (72%) cases in the database associated with type 2 poliovirus.

### Survival Analysis

The length of excretion was calculated for all 101 iVDPV cases, of which 94 (93%) were prolonged excretors and 7 (7%) were chronic excretors. At the time of analysis, 48/101 (48%) individuals had died and 36/101 (36%) had stopped excreting. There were 8/101 (8%) individuals who were still alive and excreting at last specimen and 9/101 (9%) were lost to follow-up (Table 1).

**Figure 3:**
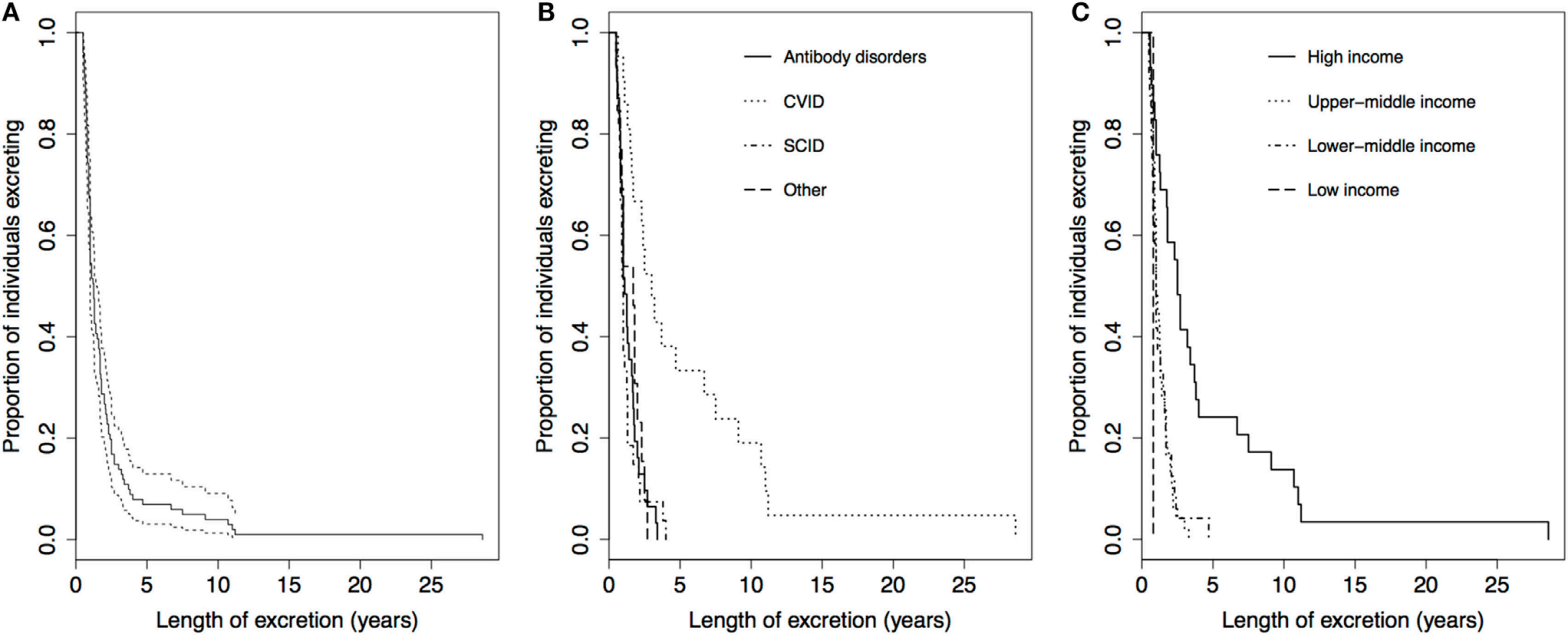
Kaplan–Meier curves for the length of poliovirus excretion in reported chronic and prolonged immunodeficiency-related vaccine-derived poliovirus cases, 1962–2016. **(A)** For all reported cases (*n* = 101, dotted lines: 95% Confidence Limits). **(B)** Comparison by income classification of country of residence: low income (*n* = 1), lower-middle income (*n* = 24), upper-middle income (*n* = 47), and high-income (*n* = 29). Two-tailed *P* < 0.001 for log-rank test for equality of Kaplan–Meier curves. **(C)** Comparison by primary immunodeficiency disorder: antibody disorders (*n* = 31), CVID (*n* = 21), SCID and other combined humoral T-cell deficiencies (*n* = 27), and other disorders (*n* = 13). Two-tailed *P* < 0.001 for log-rank test for equality of Kaplan–Meier curves. Abbreviation: CVID, common variable immunodeficiency disorder; SCID, severe combined immunodeficiency disorder.

The median length of excretion for all 101 cases was 1.3 years [95% confidence interval (CI): 1.0–1.4], with a range of 0.5–28.6 years. Figure 3 shows the Kaplan–Meier survival curves for time to cessation of poliovirus excretion, stratified by PID and country income classification. After 2.4 and 3.7 years, 80 and 90% of individuals had stopped excreting, respectively (Figure 3A). There was a significant difference in the Kaplan–Meier curves for length of excretion between different PIDs (*P* < 0.001), with individuals with CVID having the longest length of excretion, a median of 3.0 years (95% CI: 1.6–6.7) (Figure 3B). There was also a highly significant difference between different income classification of countries (*P* < 0.001), with a longer median length of excretion for high-income countries (2.5 years, 95% CI: 1.3–3.7) than upper-middle income countries (1.0 year, 95% CI: 0.9–1.3), lower-middle income countries (1.0 years, 95% CI: 0.8–1.5), and low-income countries (0.8 years) (Figure 3C). No statistical significance was found in the Kaplan–Meier curves for sex (*P* = 0.68) or poliovirus serotype (*P* = 0.21) (results not shown).

### Evolution of Length of Excretion

To understand the risk of iVDPV2 to the communities, we stratified the length of excretion by the date of onset (Table 2). Only those cases that were known to have stopped excreting (outcome alive and stopped excreting or dead) were included in this analysis to ensure comparability of the length of excretion between the two time periods 1962–2010 and 2011–2016. There was a decrease in the median length of excretion from cases with onset in 1962–2010 to 2011–2016: 1.75 years (95% CI: 1.3–2.5) and 1.0 years (95% CI: 0.8–1.4), respectively. The difference in length of excretion for the two time periods was found to be highly statistically significant when all serotypes (*P* < 0.001) were considered and specifically for type 2 (*P* < 0.001).

**Table 2:** Kaplan–Meier Estimates of the length of excretion for 84 prolonged and chronic immunodeficiency-related vaccine-derived poliovirus cases with outcome dead or stopped excreting, stratified by date of onset, 1962–2016.

Serotype^a^	Total number of cases	Date of onset 1962–2010			Date of onset 2011–2016			*P-*value^c^
		Number of cases	Median length of excretion, years	95% CI	Number of cases	Median length of excretion, years	95% CI	
1	13	9	3.20^b^		4	0.96^b^		0.002
2	54	27	1.50	1.1, 2.3	27	0.82	0.8, 1.0	<0.001
3	12	3	1.25^b^		9	1.30^b^		0.24
All	84	41	1.75	1.3, 2.5	43	1.00	0.8, 1.4	<0.001

*CI, confidence interval*.

*^a^Serotype of most recent specimen available*.

*^b^No upper CI*.

*^c^P-value for log-rank test for difference in Kaplan–Meier survival curves; tests of significance were two-sided*.

## Discussion

Our analyses document the changing epidemiology of iVDPV cases since the turn of the millenium, when the incidence of reported cases dramatically increased. The cases reside primarily from middle-income countries and are concentrated in the Middle East and North Africa. We confirm that the preponderance of cases are excreting poliovirus type 2, and demonstrate that the length of virus excretion has decreased in recent years. We also estimated that the last reported chronic excretor (greater than 5 years of excretion) had onset in 1998 and, finally, identified CVID as the major risk factor for chronic excretion.

The documented increase in number of reported iVDPV cases since the year 2000 was consistent with previous reports (17) and could be attributed to better case ascertainment, improved health care allowing immunodeficient patients to survive longer, or a combination of these factors. The surveillance sensitivity for AFP has risen in parallel with case incidence since 2000; however, this system is designed to capture cases with paralytic manifestations only (28), including those with PID. During the same period, capabilities of PID diagnostics have been enhanced and may now be available in many low- and middle-income countries. Therefore, it is possible that the increase in iVDPV cases is a surveillance artifact.

The shift in iVDPV cases from high-income to middle-income countries likely reflects two different trends: (1) high-income countries have changed their immunization schedules and switched to exclusive inactivated poliovirus vaccine use (thus preventing new iVDPVs from being generated); (2) middle-income countries have established increasingly sensitive surveillance for AFP, which better captures PIDs with paralysis; and (3) health systems in middle-income countries provide better medical care for PIDs, allowing these cases to be diagnosed and treated. However, this shift in case distribution causes new challenges to polio eradication.

The striking concentration of iVDPV cases in the Middle East and North Africa may be due to: (1) an increased risk of PID due to co-sanguinity (29) and (2) an interest of immunologists in these Regions to strengthen surveillance for PID and create immunodeficiency registries. In particular, iVDPV surveillance projects have taken place in Egypt (30), Iran (31), and Tunisia (20).

Our analysis is consistent with previous studies documenting that iVDPV type 2 constitutes more than 70% of all iVDPV cases (17), including a case that has excreted poliovirus type 2 for almost 30 years (27). Since the removal of Sabin type 2 from OPV was implemented in April 2016, no further Sabin type 2 are being introduced into populations, except for outbreak control (13, 32), which should prevent the generation of almost all new iVDPV type 2 cases.

On average, the duration of excretion of poliovirus among iVDPV cases is relatively short (approximately 1 year) and has further declined in recent years. This declining length of poliovirus excretion for all serotypes, but especially type 2, is likely to be associated with the shift in cases from high to middle income countries. This may be caused by shorter survival of iVDPV cases in middle-income countries or a higher likelihood of spontaneous cessation of excretion. However, we have little evidence to support the latter assumption.

We also document that chronic excretors are all associated with CVID in our case series. This is not surprising since CVID consists of a variety of underlying pathologies associated with later diagnosis and longer survival than some of the other PIDs diagnoses (15, 33). What is more surprising is that all chronic excretors originated from high-income countries, and that no new chronic cases have been detected since 2009. Whether this is a reflection of the quality of the health systems in developing countries to both detect and care for PID patients remains an open question.

In our case series, only eight iVDPV cases were documented to be alive and excreting. The rest either had a fatal outcome or spontaneously stopped excretion. An additional nine cases were lost to follow-up or had no information on excretion. However, if we apply the observed mortality to these cases, very few would be expected to be alive and excreting. Therefore, the known prevalence of actively excreting iVDPV cases (i.e., those captured by current surveillance systems) is small.

In terms of limitations, our analyses could only focus on those iVDPV cases reflected in the WHO registry. Our study does not address the unknown number of PIDs who excrete poliovirus but have not been captured by the AFP surveillance system because they are not paralyzed and did not come to the attention of immunologists because of either a lack of suspicion or specialist immunological services are not available. This reflects the broader challenge of tracking iVDPV cases and limitations of registries in the absence of routine screening of PID patients for poliovirus infection and excretion. Furthermore, the information available in the registry is limited by the quality of reported data. In the calculation for length of excretion, cases that were still excreting or lost to follow-up were censored at the time of last positive sample and may continue to excrete for longer time periods. Furthermore, the analysis assumed a standard evolution rate of 1.1% mutations in VP1 region of the poliovirus genome per year.

Our results have important implications for the polio endgame. The withdrawal of Sabin type 2 poliovirus in April 2016 went well (32), and future immunity to type 2 poliovirus will be induced solely by inactivated poliovirus vaccine. Our duration of excretion analysis suggests that the highest risk period for iVDPV cases re-seeding communities is in the next 2–3 years.

However, there are ways in which the Global Polio Eradication Initiative can actively decrease the risk of iVDPV cases. Generating effective therapeutic options for clearing poliovirus infections is critical. Progress has been made in this field over the last decade or more, and antiviral drugs are nearing practical applicability (34). The first drug, a capsid inhibitor, is now ready for deployment under an Investigational New Drug protocol (35). Because of high levels of drug resistance, a second drug is needed for a combination treatment (35) and related studies with a protease inhibitor are in progress (36). These drugs, as well as specific high-titer monoclonal antibodies, should provide effective treatment for iVDPV excretors. In this context, identifying PIDs with poliovirus to enable treatment with these new drugs becomes an urgent priority, not only for public health but also because these patients are at risk of developing paralytic poliomyelitis, which has a high mortality burden.

In parallel, we need to establish sensitive surveillance systems to identify and report iVDPV cases with or without paralytic symptoms, which constitute the last remaining sources of poliovirus type 2. Recent modeling by Tebbens et al. has shown that treating iVDPVs with antiviral drugs alone will have limited impact and requires integration with expanded surveillance (15). This surveillance system would be complementary to the AFP surveillance system that captures children with paralytic manifestations. The new system would also target children with PIDs (without paralytic manifestations) and would use the 10 Warning Signs of the Jeffrey Modell Foundation as a screening case definition (37). Children meeting at least two signs would then be included in the AFP surveillance system and followed up accordingly (with two stool samples collected and related laboratory investigations). The Strategic Advisory Group of Experts on Immunization Polio Working Group has endorsed this approach, and pilot country studies are being established (38). Furthermore, analysis suggests that extended surveillance could save between US$0.7 to 1.5 billion and identify 25–90% of asymptomatic iVDPV excretors (16).

This dual approach of developing therapeutic options to clear poliovirus infection and establishing sensitive surveillance for PIDs should further quantify the risks and would allow the Global Polio Eradication Initiative to actively mitigate these risks, so that global polio eradication cannot be undone by few iVDPV cases that may inadvertently re-seed communities with poliovirus, potentially leading to re-establishment of endemic or epidemic transmission. With these enhancements to the surveillance and treatment system for PIDs, we can make the final steps to a world free of polio.

## Author Contributions

Participated in research design: GM, YL, MT, OM, OK, and RS. Collected data: GM, YL, MT, KD, OM, and RS. Performed data analysis: GM, MT, YL, and SG. Wrote or contributed to drafting of early manuscripts: GM, YL, MT, KD, and RS. Critically revised manuscript: GM, YL, MT, KD, SG, OM, OK, and RS.

## iVDPV Working Group

Ousmane Diop (WHO, Switzerland), Nicksy Gumede Moeletsi (WHO Regional Office for Africa, Congo), Raffaella Williams (National Institute for Communicable Diseases, South Africa), Mohamed Seghier (Institut Pasteur d’Algérie, Algeria), Francis Delpeyroux (Institut Pasteur, France), Gloria Rey Benito (WHO Regional Office for America, USA), Maria Cecilia Freire (Instituto Nacional de Endemedades Infecciosos, Argentina), Cara Burns (Centers for Disease Control, USA), Humayun Asghar (WHO Organization Regional Office for Eastern-Mediterranean, Egypt), Salman Sharif (National Institute of Health, Pakistan), Jagadish Deshpande (Enterovirus Research Center, India), Shohreh Shahmahmoodi (Tehran University of Medical Sciences, Iran), Henda Triki (Institut Pasteur de Tunis, Tunisia), Laila E Bassioni (Egyptian Organization for Biological and Vaccine Production (VACSERA), Egypt), Amina Al-Jardani (Central Public Health Laboratory, Oman), Eugene Gavrilin (WHO Regional Office for Europe, Denmark), Merav Weil (Central Virology Laboratory, Israel), Javier Martín (National Institute for Biological Standards and Control, UK), Sirima Pattamadilok (WHO Regional Office, India), Sunethra Gunasena (Medical Research Institute, Sri Lanka), Yan Zhang (WHO Regional Office for Western Pacific, Philippines), Wenbo Xu (Chinese Center for Disease Control and Prevention, China).

## Conflict of Interest Statement

The authors declare that the research was conducted in the absence of any commercial or financial relationships that could be construed as a potential conflict of interest.

## Abbreviations

AFP, acute flaccid paralysis; CVID, common variable immunodeficiency; iVDPV, immunodeficiency-related vaccine-derived poliovirus; OPV, oral poliovirus vaccines; PID, primary immunodeficiency disorder; VDPV, vaccine-derived poliovirus; WHO, World Health Organization.
