# Supplementary material for: Prolonged Excretion of Poliovirus among Individuals with Primary Immunodeficiency Disorder: An Analysis of the World Health Organization Registry
Source: Front Immunol. 2017 Sep 25;8:1103. doi: 10.3389/fimmu.2017.01103 (PMC5622164; doi:10.3389/fimmu.2017.01103)
Supplement: Supplementary file 1 [file table_1.docx]

Table 1. Algorithm for Estimating Length of Excretion

| Information Available | | | | | Length of Excretion | Caveat | Exceptions |
| --- | --- | --- | --- | --- | --- | --- | --- |
| DOB | Date of OPV | Date of First iVDPV Isolation | Date of Most Recent iVDPV Isolation | VP1 Divergence |  |  |  |
| 🗸 | X | 🗸 | 🗸 | 🗸 | % VP1 divergence/1.1^a^ |  | If duration exceeds age of patient, then assume patient infected at birth. If duration is shorter than excretion time under observation, the latter takes precedent. |
| 🗸 | 🗸 | 🗸 | 🗸 | 🗸 | Interval between OPV and last iVDPV isolation | 1) If individual is <5 years old at onset, use date of 1^st^ OPV received. If patient is >5 years old, use date of last OPV received  2) If VP1 divergence suggests significantly shorter or longer duration, VP1 divergence takes precedent | If VP1 divergence exceeds age of patient, then assume patient infected at 1^st^ OPV |
| 🗸 | 🗸 | 🗸 | 🗸 | X | Interval between OPV and last iVDPV isolation | If individual is <5 years old at onset, use date of 1^st^ OPV received. If patient is >5 years old, use date of last OPV received |  |
| 🗸 | X | 🗸 | 🗸 | X | Interval between initial and most recent iVDPV isolation |  |  |

Abbreviations: DOB, date of birth; iVDPV, immunodeficiency-related vaccine-derived poliovirus; OPV, oral poliovirus vaccine; VP1, viral protein 1.

^a^Based on a genome evolution rate of 1.1% mutations per year
