# Supplementary material for: Prolonged Excretion of Poliovirus among Individuals with Primary Immunodeficiency Disorder: An Analysis of the World Health Organization Registry
Source: Front Immunol. 2017 Sep 25;8:1103. doi: 10.3389/fimmu.2017.01103 (PMC5622164; doi:10.3389/fimmu.2017.01103)
Supplement: Supplementary file 2 [file table_2.docx]

Supplementary Table 2. Specimen Data

| Year Detected | Country | Age^a^ (Years) | Gender | First Positive Specimen | | | Most Recent Positive Specimen | | |
| --- | --- | --- | --- | --- | --- | --- | --- | --- | --- |
|  |  |  |  | Date of Specimen | Serotype | Maximum VP1 Divergence (%) | Date of Specimen | Serotype | Maximum VP1 Divergence (%) |
| 1962 | UK | 3 | Male |  | 1 |  |  | 1 | 2.5 |
| 1962 | UK | 20 | Female | 28/01/1962 | 3 |  | 30/10/1963 | 3 | 2.3 |
| 1977 | Japan | 1.9 | Male | 15/11/1976 | 2 |  | 03/09/1979 | 2 |  |
| 1980 | USA | 1.7 | Female |  | 2 |  |  |  |  |
| 1981 | USA | 16 | Male | 07/1981 | 1 |  | 01/1982 | 1 | 10 |
| 1986 | USA | 0.9 | Male |  |  |  |  | 2 | 2 |
| 1986 | USA | 11 | Female | 1986 | 1 | 5.4 | 1992 | 2 | 11.8 |
| 1987 | UK | 34 | Male | 07/1987 | 2 |  |  | 2 | 4.1 |
| 1989 | USA | 0.6 | Female |  | 1 |  |  | 1 | 1.1 |
| 1990 | Germany | 7 | Male | 10/1990 | 1 | 5.4 | 04/1996 | 1 | 8.3 |
| 1990 | USA | 1.3 | Female |  | 2 |  |  | 2 | 1.8 |
| 1991 | USA | 0.7 | Female |  | 2 |  |  | 2 | 1.4 |
| 1995 | Iran | 1.42 | Female | 14/06/1995 | 2 | 2.2 | 17/06/1995 | 2 | 2.2 |
| 1995 | UK | 25 | Male | 03/1995 | 2 | 9.9 | 04/03/2015 | 2 | 17.7 |
| 1995 | USA | 0.3 | Female |  | 2 |  |  | 2 | 2.2 |
| 1998 | Argentina | 3 | Male | 09/1998 | 1 | 2.8 |  | 1 |  |
| 2000 | Germany | 24 | Female | 2000 | 1 | 3.5 | 2008 | 1 | 12.1 |
| 2000 | Italy | 1.67 | Female | 27/04/2000 | 2 | 0.9 |  | 2 |  |
| 2000 | UK | 13 | Male |  | 2 |  | 15/08/2005 | 2 | 7.42 |
| 2001 | China |  |  | 28/07/2001 | 3 | 1 | 29/07/2001 | 3 | 1 |
| 2001 | Taiwan | 8 | Male | 04/2001 | 1 | 1.2 | 03/2002 | 1 | 3.5 |
| 2002 | Kazakhstan | 2 | Female |  | 2 | 1.5 |  | 2 | 2.3 |
| 2002 | Kuwait | 2 | Female |  |  |  |  | 2 | 2 |
| 2002 | UK | 1.5 | Female |  | 2 |  |  | 2 | 2.5 |
| 2003 | Peru | 0.8 | Male |  | 2 | 1.2 |  |  |  |
| 2003 | Thailand | 1.5 | Male | 11/04/2003 | 2 | 1.6 |  | 2 | 2.2 |
| 2005 | China | 2 | Male | 02/08/2005 | 2 | 1.2 | 24/10/2006 | 2;3 | 4.2; 3.9 |
| 2005 | Iran | 0.6 | Male | 10/08/2005 | 2 | 1.1 | 30/11/2005 | 2 | 1.5 |
| 2005 | Morocco | 1.2 | Male | 21/07/2005 | 2 | 1.8 | 20/01/2006 | 2 |  |
| 2005 | Saudi Arabia | 0.8 |  |  | 2 | 1.9 |  | 2 | 4.4 |
| 2005 | Syria | 0.5 | Female |  |  |  |  | 2 | 1.3 |
| 2005 | USA | 0.6 | Female | 27/08/2005 | 1 | 2.3 | 01/2006 | 1 | 2.3 |
| 2006 | Iran | 0.83 | Male | 21/11/2006 | 2 | 1.7 | 24/11/2006 | 2 | 2 |
| 2006 | Iran | 1.25 | Male | 18/12/2006 | 3 | 2 | 21/12/2006 | 3 | 2.1 |
| 2006 | Syria | 0.7 | Male | 09/07/2006 | 2 | 2.2 |  |  |  |
| 2006 | Tunisia | 0.9 | Male |  | 2 | 2 |  | 2 | 2 |
| 2007 | Belarus | 3 | Male |  |  |  |  | 2 | 1.9 |
| 2007 | Iran | 0.42 | Female | 10/03/2007 | 1;2 | 2; 1.7 | 12/03/2007 | 1;2 | 2; 1.7 |
| 2007 | Iran | 0.6 | Male | 24/05/2007 | 2 |  | 14/05/2008 | 2 | 1.2 |
| 2007 | Kuwait | 0.67 | Female | 13/01/2007 | 3 | 1.2 |  |  |  |
| 2009 | Argentina | 1.3 | Male |  |  |  | 05/2009 | 1 | 3.7 |
| 2009 | Colombia | 1.3 | Male | 10/07/2009 | 2 | 1.5 | 10/07/2009 | 2 | 1.5 |
| 2009 | India | 11 | Male | 12/09/2009 | 1 | 4.1 |  | 1 | 5.2 |
| 2009 | Tunisia | 7.3 | Male | 28/11/2009 | 1 | 1.6 | 08/03/2010 | 1 | 1.2 |
| 2009 | USA | 44 | Female | 02/2009 | 2 | 12.3 |  |  |  |
| 2010 | Algeria | 0.5 | Female | 17/05/2010 | 2 | 1 | 03/10/2010 | 2 | 2 |
| 2010 | India | 10 | Female | 08/02/2010 | 2 | 1.2 | 18/03/2010 | 2 | 1.6 |
| 2010 | Iraq | 0.7 | Male | 16/12/2010 | 2 | 1.2 | 16/12/2010 | 2 | 1.2 |
| 2010 | Sri Lanka | 0.8 | Male | 07/2010 | 2 | 0.9 | 07/2010 | 2 | 1.3 |
| 2011 | Algeria | 1.2 | Female | 11/02/2011 | 3 | 2.8 | 12/02/2012 | 3 | 2.8 |
| 2011 | Algeria | 0.75 | Male | 05/04/2011 | 2 | 1 | 19/07/2012 | 2 | 2.6 |
| 2011 | China | 2.3 | Male | 18/02/2011 | 3 | 2 |  | 3 | 3.3 |
| 2011 | China | 9 | Female | 26/02/2011 | 2 |  | 28/02/2011 | 2 | 1.9 |
| 2011 | Egypt | 0.7 | Female | 03/06/2011 | 2 | 1.4 | 04/06/2011 | 2 | 1.4 |
| 2011 | Egypt | 1.7 | Male | 03/05/2011 | 1 | 2.1 | 04/05/2011 | 1 | 2.1 |
| 2011 | Egypt | 0.5 | Male | 15/07/2012 | 3 | 4.2 | 04/11/2012 | 3 | 5.3 |
| 2011 | India | 1 | Male | 18/01/2011 | 2 | 0.7 | 14/06/2011 | 2 | 0.8 |
| 2011 | India | 7 | Male | 21/10/2011 | 3 | 1.4 | 22/10/2011 | 3 | 1.2 |
| 2011 | Iran | 0.7 | Male | 23/05/2011 | 2 | 1 | 04/08/2012 | 2 | 2 |
| 2011 | Iran | 1.3 | Male | 09/06/2011 | 2 | 2.44 | 22/09/2011 | 2 | 3.77 |
| 2011 | Iran | 2.1 | Male | 16/12/2011 | 1; 2 | 2.7; 3.0 | 17/12/2011 | 1; 2 | 2.7; 3.3 |
| 2011 | South Africa | 0.8 | Male | 23/09/2011 | 3 | 1.9 | 23/09/2011 | 3 | 1.9 |
| 2011 | Sri Lanka | 8.4 | Female | 08/2011 | 3 | 1.3 | 03/2012 | 3 | 2.6 |
| 2011 | Turkey | 1 | Male |  |  |  | 13/04/2011 | 2 | 1.8 |
| 2011 | West Bank and Gaza Strip | 1 | Male | 09/11/2011 | 2 | 1.2 | 09/11/2011 | 2 | 1.2 |
| 2012 | China | 0.9 | Male | 23/02/2012 | 2;3 |  | 09/04/2012 | 2;3 | 1.3; 1.6 |
| 2012 | Egypt | 0.4 | Male | 04/12/2012 | 2 | 1 | 05/12/2012 | 2 | 1 |
| 2012 | India | 0.5 | Female | 13/02/2012 | 2 | 1.7 | 04/08/2012 | 2 | 2 |
| 2012 | Iran | 0.5 | Male | 04/03/2012 | 2 | 2.1 | 18/04/2012 | 2 | 2.4 |
| 2012 | Iran | 1 | Male | 21/08/2012 | 2 | 1.4 | 25/08/2012 | 2 | 1.5 |
| 2012 | Iraq | 2 | Male | 13/11/2012 | 2 | 1 |  | 2 | 2.4 |
| 2013 | Afghanistan | 3 | Male | 07/11/2013 | 2 | 0.9 | 08/11/2013 | 2 | 0.9 |
| 2013 | Algeria | 0.4 | Female | 12/03/2013 | 2 | 0.67 | 15/04/2013 | 2 | 1.5 |
| 2013 | China | 0.6 | Male | 30/05/2013 | 3 | 1.3 | 13/03/2014 | 2;3 | 0.6;2.1 |
| 2013 | Egypt | 0.5 | Female | 21/12/2013 | 2 | 1.3 | 19/02/2014 | 2 |  |
| 2013 | India | 0.6 | Male | 01/09/2013 | 2 | 1.1 | 27/01/2014 | 2 | 0.9 |
| 2013 | India | 0.8 | Male | 13/05/2013 | 2 | 0.9 | 06/06/2013 | 2 | 0.7 |
| 2013 | India | 0.9 | Male | 15/07/2013 | 2 | 0.7 |  |  |  |
| 2013 | Iran | 1.1 | Male | 24/01/2013 | 2 | 0.9 | 25/01/2013 | 2 | 0.9 |
| 2013 | Libya | 0.4 | Female | 05/11/2013 | 2 | 0.9 | 07/02/2014 | 2 | 1 |
| 2013 | Saudi Arabia | 2.5 | Female | 02/05/2013 | 2 | 4 | 28/08/2013 | 2 | 4.4 |
| 2013 | USA | 0.6 | Male | 07/07/2013 | 1 | 1.3 |  |  |  |
| 2014 | Albania | 0.4 | Male |  | 3 | 1.0 |  |  |  |
| 2014 | China | 1.2 | Male | 25/11/2014 | 3 | 1.4 |  |  |  |
| 2014 | Iran | 0.8 | Male | 01/06/2014 | 1 | 1.8 | 02/08/2014 | 1 | 1.8 |
| 2014 | Iran | 0.7 | Male | 06/06/2014 | 2 | 0.6 | 13/09/2014 | 2 | 0.7 |
| 2014 | Iran | 0.8 | Male | 13/02/2014 | 1 | 2.44 | 13/04/2014 | 1 | 3.3 |
| 2014 | Turkey | 2 | Female | 05/08/2014 | 3 | 1.33 | 22/02/2015 | 3 | 1.67 |
| 2015 | Algeria | 0.75 | Male | 22/07/2015 | 2 | 1.7 |  |  |  |
| 2015 | China | 0.7 | Male | 09/03/2015 | 2 | 0.78 |  |  |  |
| 2015 | Egypt | 1 | Male | 09/12/2015 | 2 | 2.1 | 25/12/2015 | 2 | 2.1 |
| 2015 | India | 2 | Female | 03/10/2015 | 2 | 2.4 | 08/03/2016 | 2 | 3.6 |
| 2015 | Iran | 0.5 | Female | 19/09/2015 | 2 | 0.9 | 07/02/2016 | 2 |  |
| 2015 | Iran | 1 | Female | 13/10/2015 | 2 | 1 | 14/10/2015 | 2 | 1 |
| 2015 | Iran | 1 | Male | 04/11/2015 | 2 | 1.8 | 08/02/2016 | 2 |  |
| 2015 | Iran | 0.8 | Female | 03/09/2015 | 2 | 1.2 | 16/02/2016 | 2 | 1.6 |
| 2015 | Iraq | 0.75 | Female | 23/07/2015 | 2 | 1.7 |  |  |  |
| 2015 | Oman | 0.7 | Male | 06/11/2015 | 2 |  |  |  |  |
| 2015 | West Bank and Gaza Strip | 0.5 |  | 02/10/2015 | 2 | 1 |  |  |  |
| 2016 | Egypt | 1 | Male | 17/04/2016 | 2 | 0.8 |  |  |  |
| 2016 | India | 5.3 | Male | 28/02/2016 | 2 | 0.6 | 29/02/2016 | 2 | 0.6 |

Abbreviations: VP1, viral protein 1.

^a^ Age of individual at date of onset or first positive specimen.

This data is contained in the World Health Organisation iVDPV Registry and is owned by the respective laboratories that generated the sequences. The data is not publicly available but will be deposited in a public repository in the future.
